# Femoral neck system versus multiple cannulated screws for the fixation of Pauwels classification type II femoral neck fractures in older female patients with low bone mass

**DOI:** 10.1186/s12891-024-07179-6

**Published:** 2024-01-13

**Authors:** Soon-Chin Yeoh, Wen-Tien Wu, Cheng-Huan Peng, Ting-Kuo Yao, Chia-Ming Chang, Kuan-Lin Liu, Tzai-Chiu Yu, Ing-Ho Chen, Jen-Hung Wang, Kuang-Ting Yeh

**Affiliations:** 1 School of Medicine, Tzu Chi University, Hualien, Taiwan; 2 Department of Orthopedics, Hualien Tzu Chi Hospital, Buddhist Tzu Chi Medical Foundation, Hualien, 970473 Taiwan; 3 Department of Medical Research, Hualien Tzu Chi Hospital, Buddhist Tzu Chi Medical Foundation, Hualien, Taiwan; 4 Department of Medical Education, Hualien Tzu Chi Hospital, Buddhist Tzu Chi Medical Foundation, Hualien, Taiwan; 5 Graduate Institute of Clinical Pharmacy, Tzu Chi University, Hualien, Taiwan

**Keywords:** Femoral neck fracture, Elderly patients, Femoral neck system, Cannulated compression screws, Adverse events

## Abstract

**Background:**

Femoral neck fractures in older adult patients are a major concern and often necessitate surgical intervention. This study compared the clinical outcomes of 2 surgical techniques: the femoral neck system (FNS) and cannulated compression screws (CCSs).

**Methods:**

A total of 40 female patients (mean age 73.50 ± 11.55 years) with femoral neck fractures of Pauwels classification type II and receiving surgical fixation between 2020 and 2022 were enrolled. The patients were categorized into an FNS group (*n =* 12) or a CCS group (*n* = 28), and surgical duration, intraoperative blood loss, length of hospital stay, and incidence of postoperative adverse events were analyzed.

**Results:**

No significant intergroup differences in demographic characteristics were discovered. The mean surgical duration for all patients was 52.88 ± 22.19 min, with no significant difference between the groups. However, the FNS group experienced significantly higher intraoperative blood loss (*P* = 0.002) and longer hospital stay (*P* = 0.023) than did the CCS group. The incidence of osteonecrosis was higher in the CCS group, whereas the incidence of nonunion or malunion was higher in the FNS group. The surgical method did not appear to be a significant risk factor. The main risk factor for revision surgery was longer duration until the first adverse event (*P* = 0.015).

**Conclusion:**

The FNS does not appear to provide superior surgical outcomes compared with CCSs in older adult women with Pauwels classification type II femoral neck fractures. A longer duration between surgical fixation and the first adverse event before stabilization of the fracture site may be a risk factor for revision surgery.

## Introduction

Femoral neck fractures are a serious public health concern, especially in older adults, due to their association with increased rates of morbidity and mortality. These fractures are also associated with reduced mobility, diminished quality of life, and increased risk of complications [1, 2]. Various surgical techniques have been developed and refined to optimize clinical outcomes in the treatment of femoral neck fractures. Two commonly used techniques are the use of the femoral neck system (FNS) and the use of cannulated compression screws (CCSs). CCSs are widely employed due to their technical simplicity, minimal invasiveness, and cost-effectiveness [3]. However, studies have indicated potential limitations of CCSs, such as suboptimal biomechanical stability resulting in nonunion and avascular necrosis of the femoral head [4, 5]. The FNS is a more modern technology than is the CCS; the FNS has superior biomechanical properties that increase the likelihood of fracture union and reduce the likelihood of complications [6, 7]. Advancements have continued in orthopedic techniques and technologies; however, a comprehensive and analytical comparison of the FNS with CCSs, particularly a comparison that focuses on clinical outcomes in older adult patients, has not yet been conducted. Older adult patients present a unique set of challenges, given their increased likelihood of osteoporosis and other illnesses and their relatively low physiological reserves; the choice of fixation method is thus critical in these patients [8, 9].

This study compared the efficacy of the FNS and CCSs for the treatment of femoral neck fractures in older adults. The findings of this study contribute to our understanding of the optimal treatment protocol for femoral neck fractures and can ultimately reduce the morbidity and mortality associated with femoral neck fractures in this vulnerable demographic. Through an evidence-based approach, this study highlighted the advantages and limitations of both fixation methods, providing guidance to clinicians when making decisions related to the management of femoral neck fractures in older adult patients.

## Materials and methods

This was a retrospective cohort study. Data were extracted from multiple health-care databases. The clinical outcomes achieved using the FNS versus CCSs for the fixation of femoral neck fractures in patients aged 60 years or older were compared. Ethical approval was obtained from the Ethics Committee of the Institutional Review Board of Hualien Tzu Chi Hospital, Buddhist Tzu Chi Medical Foundation. Patient consent was waived due to the retrospective nature of the study. All data were anonymized to maintain confidentiality.

**Fig. 1:**
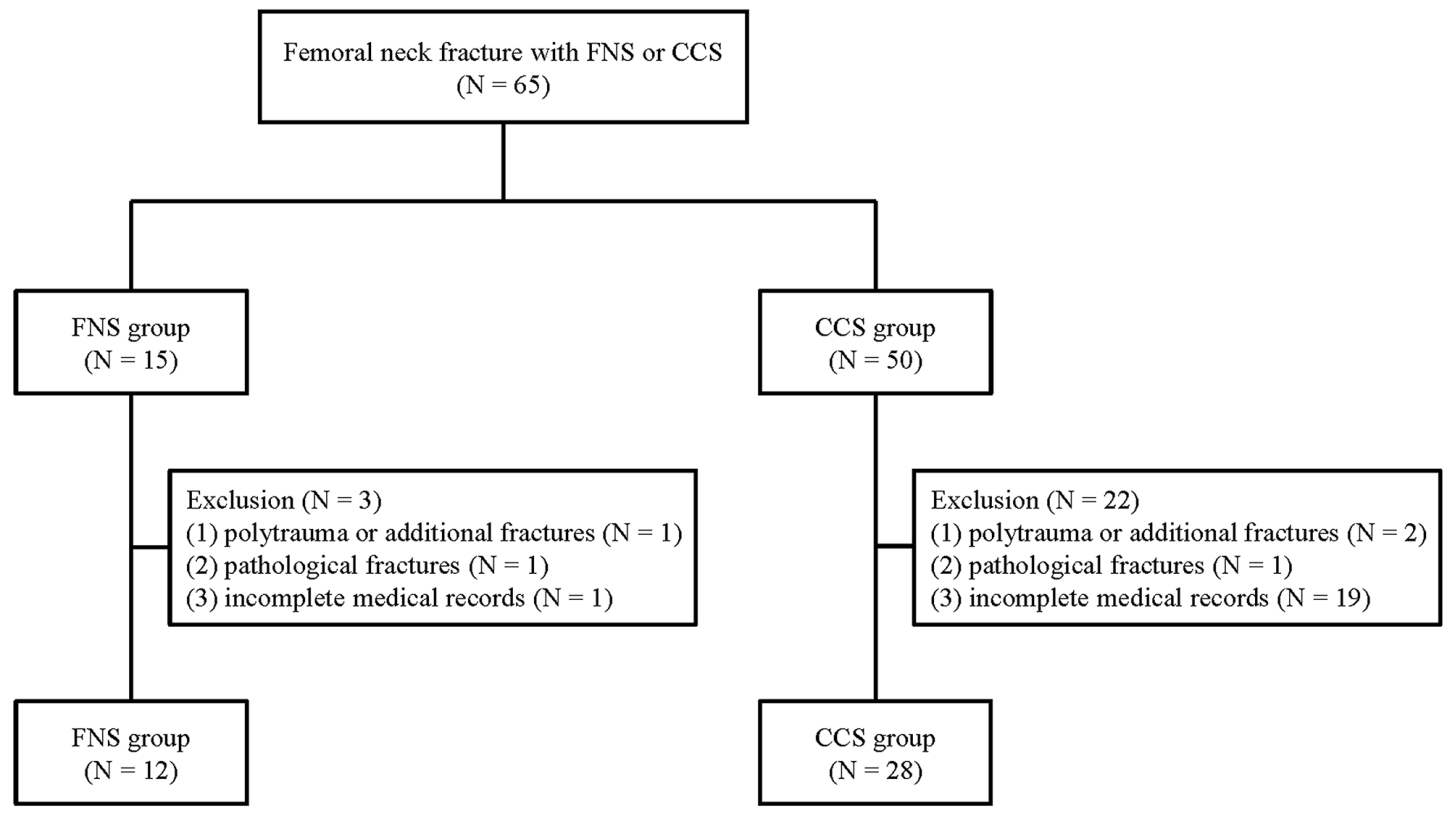
The flowchart of this study

The study population comprised patients aged 60 years or older with a diagnosis of femoral neck fracture of Pauwels classification type II who received surgical fixation using the FNS or CCSs in a single eastern Taiwan medical center between January 2020 and June 2022. Patients were excluded if they had (1) a history of polytrauma or additional fractures affecting the outcomes, (2) pathological fractures, or (3) incomplete medical records. Patients were assigned to the FNS group if they underwent femoral neck fracture fixation using the FNS (DePuy-Synthes, Zuchwil, Switzerland), or to the CCS group if their fixation using the 3 or 4 cannulated compression screws (Fig. 1).

Patients received treatment within 24 h of receiving a diagnosis of fracture. Surgeries were performed by 5 orthopedic surgeons who each had at least 5 years of experience in trauma surgery. All patients underwent the same rehabilitation program, which consisted of the following: (1) mobilization in bed in postoperative day 1; (2) quadriceps femoris exercises and both passive and active hip, knee, and ankle exercises, which were initiated on postoperative days 1–3; (3) partial weight-bearing exercises with walker frame assistance, which were initiated on postoperative days 3–7 on the basis of the patient’s recovery status; (4) near full weight-bearing exercises with walking stick assistance, which were undertaken independently by the patients in accordance with their recovery status 3–6 months after surgery. on the basis of the patient’s recovery status and bone healing progress; and (5) full weight-bearing exercises, which were initiated in postoperative months 3–6 on the basis of the patient’s recovery status and bone healing progress.

Data on demographic characteristics, preoperative health status (evaluated using the Charlson comorbidity index), perioperative parameters, and postoperative adverse events were retrospectively collected from electronic medical records. The primary outcome was the rate of postoperative complications, which were nonunion or malunion, hardware failure, femoral head osteonecrosis, and surgical site infection. This outcome is presented as the number of adverse events per patient.

### Statistical analysis

Descriptive statistics were used to analyze the data. Categorical variables were compared using chi-square tests, and continuous variables were compared using independent t tests. A stepwise multivariate linear regression analysis was performed to estimate associations between the incidence of revision surgery and various demographic and clinical parameters. *P* values of < 0.05 indicated statistical significance.

## Results

### Demographic characteristics

In total, 40 women were enrolled and categorized into the FNS group (*n* = 12) or CCS group (*n* = 28). The average age of the patients was 73.50 years (standard deviation: 11.55 years). Their average T-score for bone mineral density was − 2.90 (standard deviation: 0.60), and their mean body mass index was 22.90 kg/m^2^ (standard deviation: 5.01 kg/m^2^; Table 1). The mean Charlson comorbidity index score for the entire cohort was 3.55 (standard deviation: 1.87). No significant differences in any of the demographic characteristics were discovered between the FNS and CCS groups (Table 1).

**Table 1:** Demographics (*n* = 40)

Item	FNS	CCS	Total	***P***-value
N	12	28	40	
Age	68.08 ± 7.94	75.82 ± 12.18	73.50 ± 11.55	0.051
BMD T score	-2.83 ± 0.41	-2.93 ± 0.66	-2.90 ± 0.60	0.649
BMI (kgw/m2)	22.96 ± 4.07	22.88 ± 5.43	22.90 ± 5.01	0.962
CCI	3.58 ± 1.93	3.54 ± 1.86	3.55 ± 1.87	0.853
Surgical time (mins)	58.25 ± 13.83	50.57 ± 24.80	52.88 ± 22.19	0.322
Blood loss (cc)	166.67 ± 80.75	84.29 ± 47.05	109 ± 51.11	0.002*
LOS (days)	7.25 ± 3.57	5.00 ± 2.33	5.68 ± 2.90	0.023*
Duration to first adverse event (months)	1.56 ± 1.70	1.57 ± 3.14	1.57 ± 2.76	0.989
Adverse event number	0.83 ± 1.27	0.46 ± 0.92	0.58 ± 1.03	0.307
Nonunion / Malunion	3 (25%)	1 (3.6%)	4 (10%)	
Hardware failure	2 (16.7%)	1 (3.6%)	3 (7.5%)	
Osteonecrosis	1 (8.3%)	6 (21.4%)	7 (17.5%)	
Surgical site infection	0 (0%)	0 (0%)	0 (0%)	
Revision (%)	2(16.7%)	6(21.4%)	8(20.0%)	1.000

*BMD* bone mineral density, *BMI* body mass index, *LOS* length of stay, *FNS* femoral neck system, *CCS* conventional compression screws, *CCI* charlson comorbidity index

Data are presented as *n* or mean ± standard deviation. *P-value < 0.05 was considered statistically significant after test

### Surgical metrics and intraoperative variables

The average surgical duration for the entire cohort was 52.88 min (standard deviation: 22.19 min). The surgical duration was slightly longer in the FNS group (58.25 ± 13.83 min) than in the CCS group (50.57 ± 24.80 min); however, this difference was not significant. The mean intraoperative blood loss was greater in the FNS group (166.67 ± 80.75 cc) than in the CCS group (84.29 ± 47.05 cc; *P* = 0.002), and the mean hospital stay was longer in the FNS group (7.25 ± 3.57 days) than in the CCS group (5.00 ± 2.33 days; *P* = 0.023; Table 1).

### Postoperative complications and adverse events

The mean duration until the first adverse event was 1.57 ± 2.76 months, and the average number of adverse events per patient was 0.58 ± 1.03 (Table 1). The FNS group had 3 cases of nonunion or malunion, 2 cases of hardware failure, and 1 case of osteonecrosis, whereas the CCS group had 1 case of nonunion or malunion, 1 case of hardware failure and 6 cases of osteonecrosis. Infection did not occur in either group (Table 1). This study investigated the risk factors associated with the incidence of revision surgery. The only significant risk factor for revision surgery was longer duration between surgical fixation and the first adverse event (OR = 5.51, 95% confidence interval = 1.38–21.93; *P* = 0.015; Table 2).

**Table 2:** Risk factors associated with revision surgery (*n* = 40)

	Crude		Adjusted	
	OR (95% CI)	***P***-value	OR (95% CI)	***P***-value
Age	1.03 (0.96, 1.11)	0.355	1.25 (0.85, 1.82)	0.254
Method (FNS vs. CCS)	0.73 (0.13, 4.29)	0.731	0.42 (-0.32, 1.22)	0.272
BMD T score	0.32 (0.07, 1.44)	0.136	1.23 (0.02, 64.87)	0.919
BMI (kgw/m^2^)	0.88 (0.73, 1.08)	0.216	0.98 (0.68, 1.42)	0.922
CCI	1.23 (0.83, 1.81)	0.302	0.36 (0.06, 2.12)	0.261
Surgical time (mins)	0.98 (0.95, 1.03)	0.444	-0.03 (-0.06, 0.01)	0.082
Blood loss (cc)	1.00 (1.00, 1.01)	0.723	0.02 (-0.01, 0.04)	0.312
LOS (days)	1.07 (0.83, 1.38)	0.621	-0.03 (-0.11, 0.05)	0.411
Duration to first adverse event (months)	3.66 (1.57, 8.54)	0.003*	5.51 (1.38, 21.93)	0.015*

*BMD* bone mineral density, *BMI* body mass index, *LOS* length of stay, *FNS* femoral neck system, *CCS* conventional compression screws

Data are presented as odds ratio (95% CI). *P-value < 0.05 was considered statistically significant after test.

### Case presentation

#### Case 1 (FNS group)

**Fig. 2:**
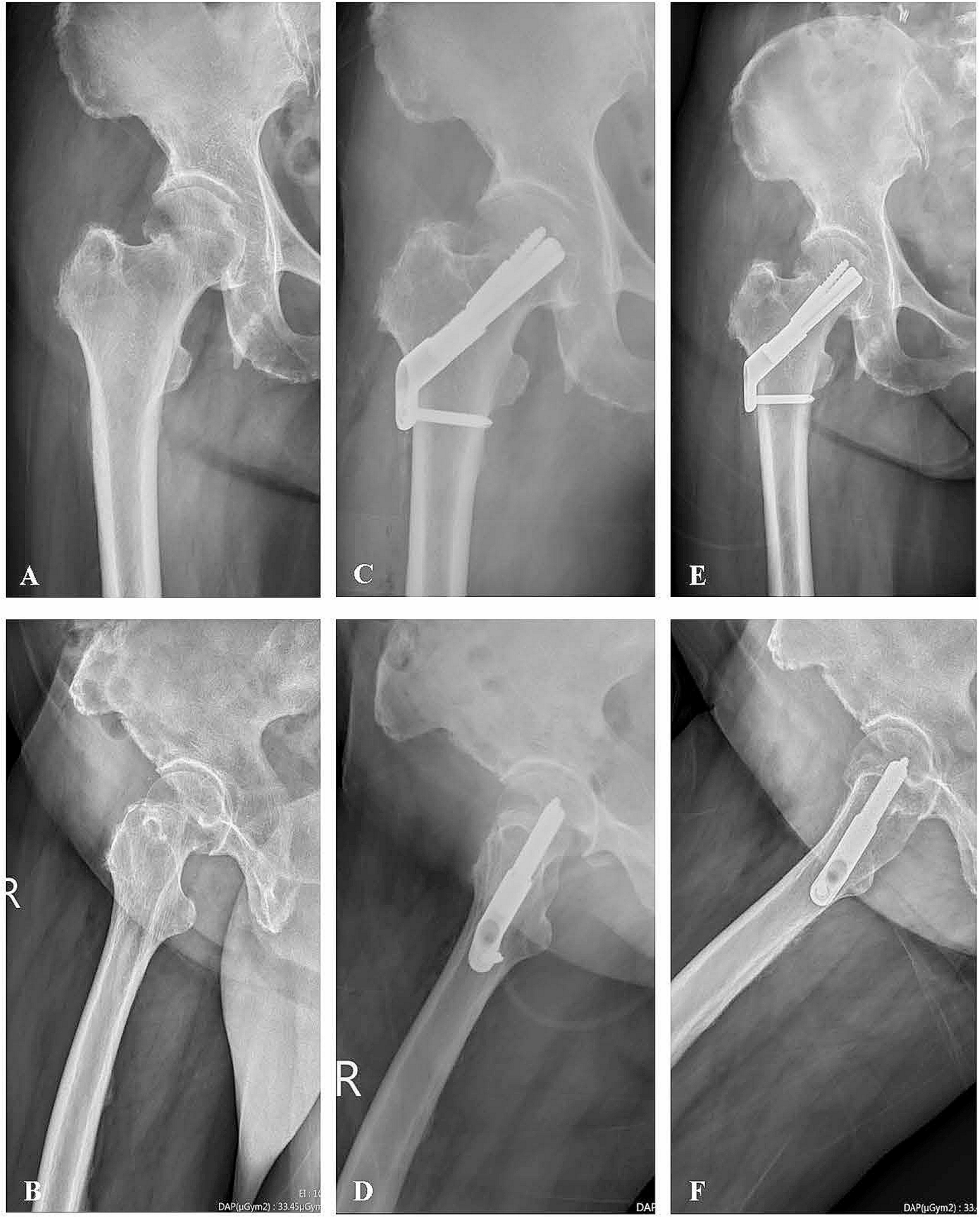
A 71 y/o woman with right transcervical femoral neck fracture (Pauwel Type II): **A** Preoperative AP view; **B** Preoperative lateral view; **C** Immediate postoperative AP view; **D** Immediate postoperative lateral view; **E** Postoperative AP view at 6 months; **F** Postoperative lateral view at 6 months

A woman aged 71 years with hyperthyroidism s/p total thyroidectomy was experiencing right hip pain after a fall. The woman was diagnosed with right transcervical femoral neck fracture (Pauwels Type II). Injury films are shown in Fig. 2A, B. Immediate postoperative radiographs indicated anatomic fracture reduction and fixation with the FNS (Fig. 2C, D). By the 6-month follow-up, the patient’s fracture had healed, and anatomic alignment had been maintained (Fig. 2E, F). The patient was able to walk without assistance at 8 months after her operation.

#### Case 2 (FNS group)

**Fig. 3:**
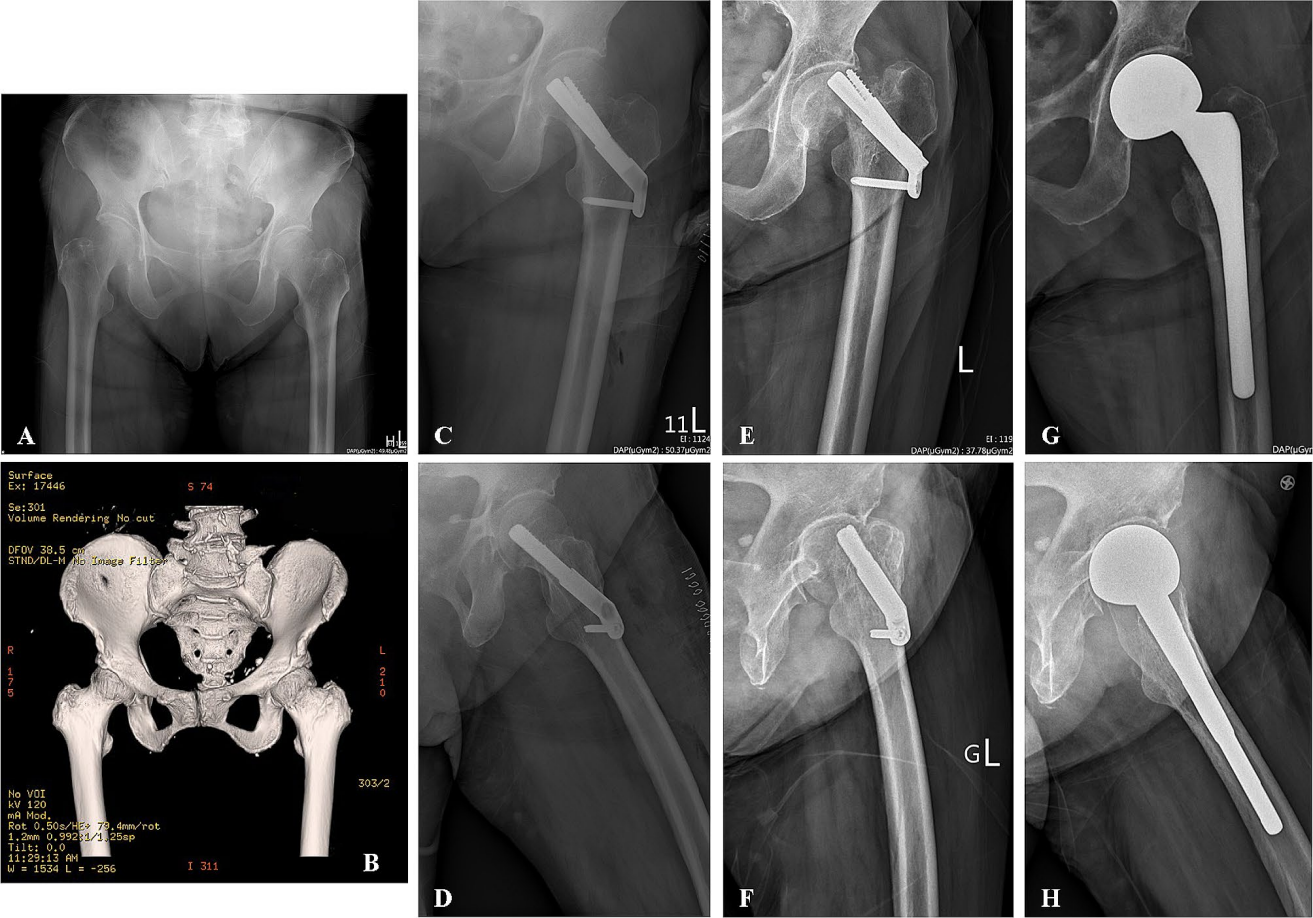
A 74 y/o woman with subcapital femoral neck fracture (Pauwel Type II): **A** Preoperative AP view; **B** Preoperative 3D reconstruction CT scan; **C** Immediate postoperative AP view; **D** Immediate postoperative lateral view; **E** Postoperative AP view at 8 months; **F** Postoperative lateral view at 8 months; **G** Post-hemiarthroplasty AP view; **H** Post-hemiarthroplasty Lateral view

A woman aged 74 years with a history of hypertension, type II diabetes mellitus, and osteoporosis was given a diagnosis of left subcapital femoral neck fracture (Pauwels Type II) after a road traffic accident (Fig. 3A, B). Immediate postoperative radiographs indicated anatomic fracture reduction and fixation with the FNS (Fig. 3C, D). However, implant cutout was required 8 months later (Fig. 3E, F). Subsequently, the implant was removed, and bipolar hemiarthroplasty was performed (Fig. 3G, H). Malunion and shortening of the femoral neck were noted during the operation. The patient was able to walk without assistance at 6 weeks after the bipolar hemiarthroplasty.

#### Case 3 (CCS group)

**Fig. 4:**
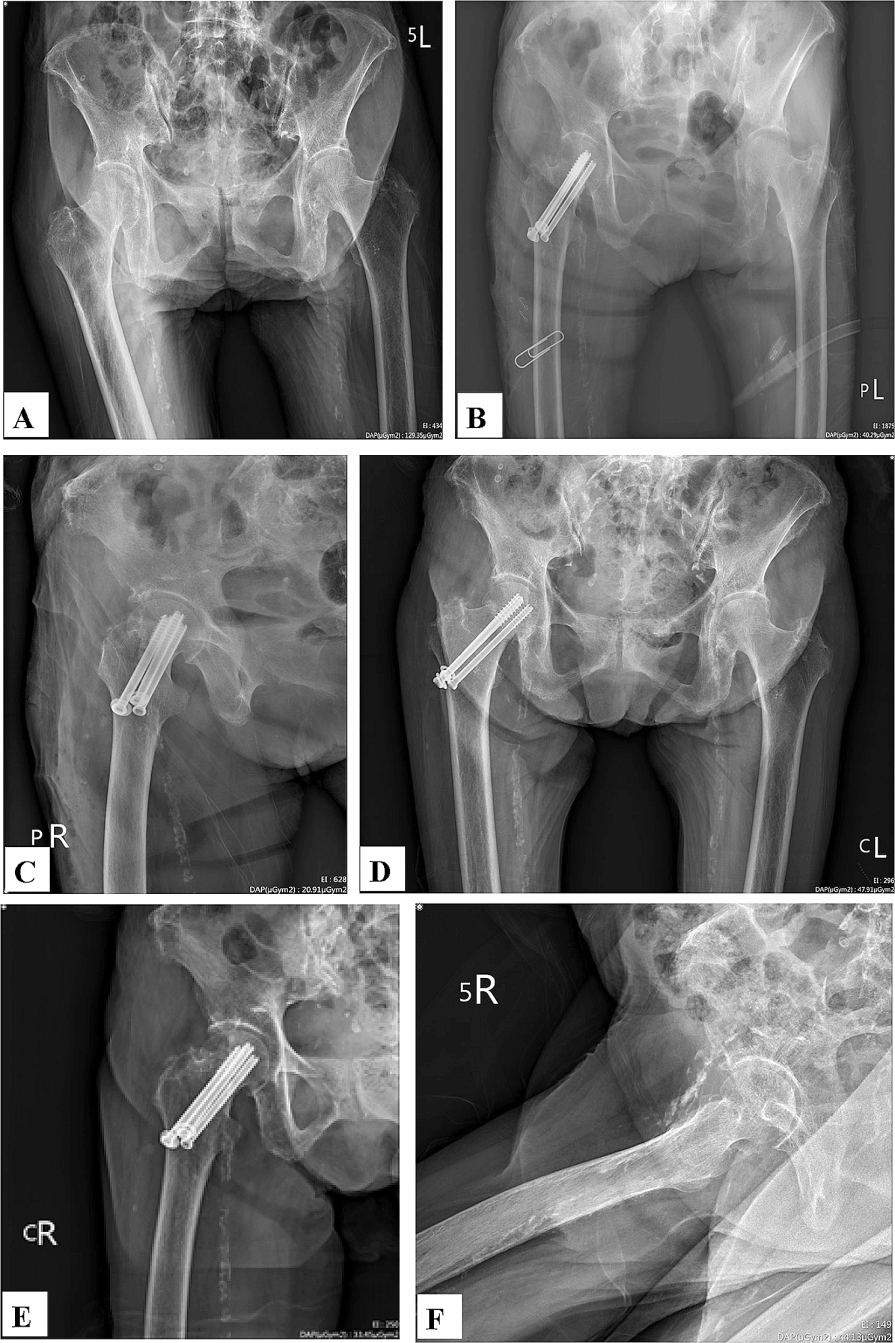
A 80 y/o woman with right transcervical femoral neck fracture (Pauwel Type II): **A** Preoperative AP view; **B** Immediate postoperative AP view; **C** Immediate postoperative lateral view; **D** Postoperative AP view at 12 months; **E** Postoperative lateral view at 12 months; **F** Postoperative AP view after removal of implants

A woman aged 80 years with a history of hypertension and osteoporosis was given a diagnosis of right subcapital femoral neck fracture (Pauwels Type II) after a fall (Fig. 4A). Immediate postoperative radiographs indicated anatomic fracture reduction and fixation with 3 CCSs (Fig. 4B, C). By the 12-month follow-up, the fracture had healed, and anatomic alignment had been maintained (Fig. 4D, E). The implant was removed in postoperative month 15 (Fig. 4F).

## Discussion

Fixation with the FNS did not appear to be superior to fixation with CCSs in our cohort of older adult women (mean age 73.50 ± 11.55 years) with Pauwels classification type II femoral neck fractures. No significant differences in demographic characteristics were found between the groups in our study. This study contributes to the limited body of English-language literature comparing the FNS with CCSs for the treatment of femoral neck fractures in older adults, acknowledging the possibility of relevant research in non-English publications. Stoffel et al. reported that the FNS is a valid alternative treatment technique for unstable femoral neck fractures and that, from the biomechanical perspective, it has comparable stability to the Dynamic Hip Screw systems and is superior to the use of CCSs [10]. Huang et al. claimed that for treating vertical femoral neck fractures, the FNS may be superior to traditional CCSs in terms of their biomechanical and clinical aspects [11]. For nongeriatric patients with femoral neck fracture (stable or unstable), the FNS was effective in improving hip function and reducing the femoral neck shortening rate and fluoroscopy exposure. The FNS was also associated with a lower incidence of complications compared with CCSs [12, 13]. However, according to a systemic review and meta-analysis conducted by Rajnish et al. the rates of various complications—such as implant failure, nonunion, and avascular necrosis—are similar between the FNS and CCSs, and neither technique is superior in terms of improvement in final functional status or pain relief [14]. Our results are consistent with the findings of that meta-analysis. In our study, greater mean intraoperative blood loss and longer hospital stays were discovered in the FNS group than in the CCS group; these factors may explain the nonsuperiority of the FNS method. Older adults are likely to have comorbidities that affect surgical outcomes, and the relative simplicity and shorter operative time associated with CCSs might make CCSs a more appropriate technique for older adults when considering the overall risk posed by a surgery [15]. In addition, in older adult women, low bone quality may undermine the mechanical advantages offered by more advanced systems such as the FNS. By contrast, CCSs, being less reliant on bone quality for stability, may perform comparably in such patients [16]. Although the FNS was designed to provide angular stability, its efficacy is partly dependent on the bone’s ability to support the implant, and this support is compromised in osteoporotic bone. Essentially, the FNS provides enhanced stability but requires sufficient bone integrity for optimal function [12]. By contrast, CCSs are less dependent on bone quality, and although they provide less stability than does the FNS, CSSs provide multidirectional fixation and remain effective even in osteoporotic bone [17]. The overall outcome in femoral neck fracture repair depends on not only mechanical stability but also the biological environment, which may be less favorable in older adults [18]. Another consideration that is often overlooked but increasingly relevant in health-care decision-making is cost-effectiveness. Due to its complex design, the FNS is more expensive than are traditional, simpler systems. Patients receiving treatment with the FNS may have smaller resources to allocate to postoperative supportive care, which appeared in one study to be as important as the surgical method in the treatment of femoral neck fractures [19].

The most notable differences in the observed outcomes between the groups in our study were the higher incidence of osteonecrosis in the CCS group and the higher incidence of nonunion or malunion in the FNS group. CCSs, although beneficial in compressing fractures, may interfere with residual blood flow to the femoral head, potentially contributing to the development of osteonecrosis [6, 20]. This risk is likely influenced by the number and arrangement of screws [21]. The FNS may mitigate the risk of vascular insult because it is a less invasive and more biomechanically stable construct [22]. The risk of nonunion or malunion, which are significant postoperative setbacks, is influenced by multiple factors, such as surgical technique, bone integrity, and implant attributes [23]. Although the FNS provides superior mechanical stability, it may paradoxically inhibit the bone-healing process by inadvertently suppressing callus formation [24, 25]. These complications are likely more prominent in older adult patients than in younger patients.

A significant risk factor for revision surgery was found to be longer duration between surgical fixation and the first adverse event. Early complications are often linked to surgical factors, whereas late complications tend to be associated with patient factors, such as underlying health conditions or rehabilitation challenges [26]. A delayed onset of the first adverse event may indicate a period of subclinical vulnerability, where unapparent issues accumulate, leading to a cascade of complications later on. This explanation is supported by research indicating that initial postoperative stability does not always preclude late complications [6]. Older adult patients, particularly those with comorbidities or poor nutritional status, may exhibit a delayed response to the initial surgical trauma, culminating in late-onset complications [27]. Additionally, extended periods without complications may encourage less stringent postoperative monitoring or adherence to rehabilitation protocols. Consistent rehabilitation and follow-up are crucial to the early detection and management of complications [21]. For older adult patients with femoral neck fracture who have undergone surgery, regular and intensive follow-up and outpatient visits may be necessary until the fracture has healed and complications at the fracture site have been ruled out.

On the basis of our findings and review of the literature, possible indications for the FNS include the following: (1) unstable or comminuted femoral neck fracture [11], (2) a history of nonunion with CCSs [13], and (3) younger patients [28]. Contraindications for the FNS include the following: (1) cost considerations [19], (2) less experienced surgeons for whom the technical complexity of the FNS may be excessive [29], and (3) older adult patients who lack the resources to obtain supportive care during their recovery [13]. The choice between the FNS or CCSs should be tailored to the individual patient’s needs, the characteristics of their fracture, and the clinical setting. Although the FNS shows promise for complex fracture patterns and demanding functional scenarios, its higher cost and technical complexity makes it less universally applicable than CCSs for older adult patients.

## Implications and limitations

This study was limited by its small and women-only sample, which may have affected the statistical power, effect size, and generalizability of the findings. Nevertheless, our findings have several clinical implications. For instance, the lack of significant differences in surgical duration between the FNS and CCS groups implies that either method can be implemented without notable alterations to a surgical workflow. However, the greater blood loss and longer hospital stay associated with the FNS may necessitate more comprehensive preoperative planning and postoperative care. To build upon the findings of this study and enhance understanding of the clinical efficacy of the FNS versus CCSs, future studies should involve larger sample sizes, more diverse populations of patients from multiple institutions, long-term follow-ups, subgroup analyses, and propensity score matching. Patient-reported outcomes and functional score data could also be analyzed.

## Conclusion

Our study provides essential insights into the relative efficacy of the FNS versus CCSs in the surgical management of Pauwels classification type II femoral neck fractures in an older adult female population with low bone mass. The FNS was not found to be superior to CCSs for fixation of femoral neck fractures in this specific cohort. The FNS may be associated with greater intraoperative blood loss and a longer hospital stay than are CCSs. The surgical method did not appear to be a significant risk factor for revision surgery; however, longer duration between surgery and the first adverse event was a risk factor for revision surgery. Regular follow-up is necessary. The present findings may serve as a reference for orthopedic surgeons making decisions on the best surgical method for treating older adult patients, especially when cost considerations are important. The findings may be limited by the study’s small sample size. Future research with larger and more diverse populations, longer follow-ups, and functional outcome collection is essential to validate and expand upon the findings, ultimately guiding clinical decision-making regarding hip fracture surgery.

## Data Availability

All data generated or analyzed during this study are included in this published article.

## Abbreviations

FNS: femoral neck system
CCS: cannulated compression screws
